# Understanding the complexity of hydrolysates

**DOI:** 10.1186/1753-6561-7-S6-P21

**Published:** 2013-12-04

**Authors:** Abhishek J Gupta, Kathleen Harrison, Dominick Maes

**Affiliations:** 1 Laboratory of Food Chemistry, Wageningen University, Wageningen, The Netherlands; 2 FrieslandCampina Domo, Delhi, NY 13753, USA; 3 FrieslandCampina Domo, Wageningen, The Netherlands

## Background

Hydrolysates are complex media supplements composed of many as well as different types of compounds. Within Frieslandcampina Domo's Quality by Design project, detailed information of these compounds (annotation and quantification) has been generated. This was achieved for soy protein hydrolysates (Proyield Soy SE50MAF-UF) using metabolomics biochemical profiling. Biochemical profiling, together with peptide profiling and analysis of the inorganic compounds, resulted in complete characterization of this hydrolysate product. Additionally, these lots of Proyield Soy SE50MAF-UF were tested for cell culture performance.

## Results and Discussion

The composition data was natural log transformed and functionality data was corrected for experiment-to-experiment variation. Consequently, the dataset was analyzed using statistical tools like two-mode cluster analysis, bootstrapped stepwise regression and 2D correlation analysis. These statistical tools were composed in-house using Matlab^® ^R 2009b version 7.9.0.529.

This resulted in identification of a series of key compounds in the hydrolysates that correlated with cell growth or IgG production in a CHO cell line. To validate these findings, pure preparations of these key compounds were supplemented to the chemically defined medium. Addition of these individual key compounds to chemically defined medium, in some cases, slightly improved cell growth or IgG production, but the effect was still much smaller than the enhancing effect of the complete hydrolysate. The specific IgG production of key compounds supplemented to CD media, CD media alone, and soy protein hydrolysate supplemented to CD media is shown in Table 1.

**Table 1:** Key compounds supplemented at 0.01% (w/v) to CD media.

Key compound	Specific IgG production (%)*
Ferulic acid	154
Syringic acid	194
Galactarate	153
Adenine	185
Trigonelline	141
SE50MAF-UF	204
CD media	100

All values are set relative to CD media (100%).

This suggests that the effect of a hydrolysate cannot by mimicked by adding certain key compounds. Alternatively, this suggests that these key compounds are biomarkers, which are interconnected with several other compounds, and that presence of all of these compounds is relevant/important for the enhancement in the functionality.

**Figure 1:**
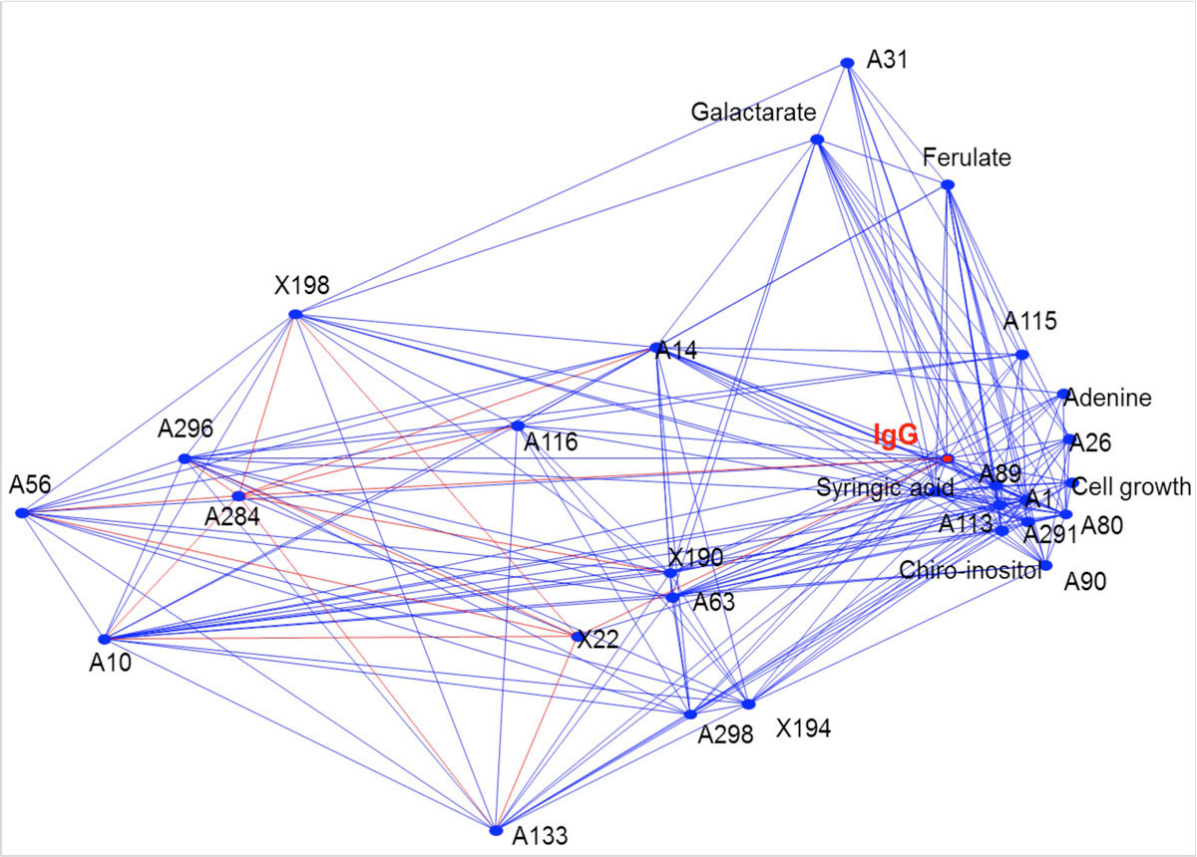
**2D correlation map of compounds present in ProYield SE50MAF-UF that significantly influences IgG production by CHO cells**. These key compounds are interconnected to other compounds of the hydrolysate, forming a complex biochemical network.

The 2D correlation analysis reveals this complex network of compounds, in which these compounds are positively or negatively correlated with each other and with cell growth or IgG production (Figure 1).

In hydrolysates, these compounds interact with several other compounds in a complex biochemical network. This network of compounds is a unique and native feature of hydrolysates and non-existent in chemically defined media.

Working in close collaboration with our customers, we gain understanding about the relation between the complex composition of hydrolysates and their effect on cell growth and titer in the application.

